# When to Watch out

**Published:** 1901-07

**Authors:** C. E. Boynton

**Affiliations:** Sandy, Utah


### CORRESPONDENCE.

### WHEN TO WATCH OUT.

*Editor Atlanta Journal-Record of Medicine:*

1. If you give 1-100 gr. glonoin to a weak patient who is sitting up, fainting, dilated pupil and pallor may follow, particularly if stomach is empty.

2. Morphine $\frac{1}{3}$ grain to patient who is frail and over 60 years of age may give trouble. Go slowly with morphine in the aged.

3. When rectal temperature touches $104\frac{1}{2}°$ at your first call, don't prognose hopefully; if fever has been at that height for some time and it is not malaria, the result may be fatal in spite of all a physician can do.

4. When a patient may be dying don't give a hypodermic precipitously, if the people are ignorant and suspicious. They may charge you with killing the patient.

5. If you have just located in a town, don't take all the old chronic cases that come to you and promise cure, even when cure might be expected, for these people are often the victims of habit and environment beyond the reach of a physician's best efforts.

6. Watch out when a sad-eyed woman with a long invalid history wants you to take her case, and you are able to find no organic lesion worth considering. Ten to one she lives an unhappy sexual life that is beyond the art of a medical man to improve.

7. Watch out for your bill when new patients praise you profusely.

8. If you are the "new doctor" and the old physician is handy, look out, or he may be called if your first prescription does not work wonders.

9. Look out for some of your pay when the patient is convalescing.

10. Look out for hemorrhage, if you have administered over 20 grains of quinine in confinement.

11. Look out that the kidneys are not too long inactive after you have used large doses of atropia.

12. See that in fever, pain or delirium, the bowels are kept antiseptic and active.

13. In enuresis or nervous derangements of children, examine the genital organs.

14. Look out for sickness in a farmer's family just after pig-killing, and have your saline laxative, oil and calomel handy.

15. Look out for the "blues," when you apply ascetanilid locally or administer it for several doses internally.

16. Look out that a sick-room does not become an assembly hall.

17. Look out in cold weather that the sick-room is well ventilated—two windows lowerd from the top. Temperature below 75° F.

18. Boil the drinking-water, if there is the remotest suspicion that it may be infected.

19. Look out that a sick child eats no bananas.

20. If the mother is alarmed, look out for the child, even though things look all right.

21. Look out if a child is quite sick and vomits with great difficulty. A sensitive vomiting center is ofttimes a safety valve.

C. E. BOYNTON, M.D.

*Sandy, Utah.*

